# Assay development for the discovery of small-molecule inhibitors of YadA adhesion to collagen

**DOI:** 10.1016/j.tcsw.2019.100025

**Published:** 2019-05-23

**Authors:** Athanasios Saragliadis, Dirk Linke

**Affiliations:** Section for Genetics and Evolutionary Biology, Department of Biosciences, University of Oslo, Oslo, Norway

**Keywords:** Bacterial adhesion, Adhesin, YadA, TAA, Assay development, HTS

## Abstract

We set out to develop scalable assays to measure bacterial adhesion to mammalian extracellular matrix proteins, with the aim to perform high-throughput screening for inhibitors. Our model system is the trimeric autotransporter adhesin YadA from *Yersinia enterocolitica* that binds to collagen.

Using bacterial cells expressing GFP under an inducible promotor, and co-expressing the adhesin of choice, we were able to establish a 384-well plate-based assay that allowed us to screen 28,000 compounds in 8 days (3520 compounds per day). We have collected all parameters that were essential in assay development, and describe how they can be tuned for improved performance.

Out of 28,000 compounds, 5 compounds showed significant inhibitory activity, measured as loss of fluorescence compared to control wells. Our assay is easy to scale up, and can be adopted to different ECM component/Adhesin combinations. Alternatively, bacterial pathogens (harboring deletion mutants of adhesins compared to wildtype) could be used directly in the same assay if they express GFP as a reporter at high levels.

## 1 Introduction

Antimicrobial resistance in bacterial pathogens is a critical problem, and in recent years, hardly any novel antibiotics to overcome this issue have been brought to market. One underlying reason for this is the inherently limited number of conserved and essential cellular targets that antibiotics aim to disrupt; namely DNA gyrase and topoisomerase IV, ribosomal functions or cell-wall biosynthesis (Payne et al., 2007). The future of antimicrobial therapy might thus lie in more targeted substances that specifically inhibit only a subset of pathogenic bacteria, combined with better (pre-treatment) diagnostics (Spaulding et al., 2018).

Bacterial adhesion is the first step of bacterial colonization, and an essential part of invasive processes as well as of biofilm formation. The proteinaceous bacterial appendages known as adhesins form an attractive target candidate for discovering novel antimicrobial compounds. One family of adhesins exclusive to Gram-negative bacteria are the trimeric autotransporter adhesins (TAAs). They are essential for the pathogenesis of a number of *Enterobacteriaceae*, including *Yersinia enterocolitica* and *Yersinia pseudotuberculosis* (Mikula et al., 2013, Sabina et al., 2011), and appear to be important in various stages of pathogenesis in entero- and uropathogenic *E. coli* (Totsika et al., 2012), *Salmonella* (Raghunathan et al., 2011), and *Proteus* (Alamuri et al., 2010). Experimental approaches as well as sequence and structure comparisons show that the domain mainly responsible for binding to extracellular matrix molecules, and specifically to collagen, is the so-called head domain of TAAs (Leo et al., 2012, Linke et al., 2006)

The *Yersinia enterocolitica* Adhesin A (YadA) is the best characterized and the archetype of TAAs. *Y. enterocolitica* is the causative agent for various diseases such as enterocolitis, acute mesenteric lymphadenitis, septicemia and metastatic infections and pharyngitis (Bottone, 1997). YadA is encoded for on the *Yersinia* virulence plasmid (pYV), together with the components of a type III secretion system (El Tahir and Skurnik, 2001, Oberhettinger et al., 2011). YadA of both *Y. enterocolitica* and *Y. pseudotuberculosis* binds to Factor H (Biedzka-Sarek et al., 2008), and is involved in processes such as autoagglutination (Skurnik et al., 1984), serum resistance (Balligand et al., 1985), adherence to and phagocytosis resistance to HEp-2 cells (Heesemann and Grüter, 1987). YadA directly binds to various ECM components, including collagens (Schulze-Koops et al., 1992), laminin (Tamm et al., 1993), and immobilized fibronectin (Tertti et al., 1992).

Inhibiting bacterial adhesion, both during infection situations (e.g. diarrhea, urinary tract infections) and as a preventive measure (e.g. on implants, catheters) is our long-term goal. To this end, we developed systematic screening assays for small molecules that inhibit adhesion either directly by competitive binding to specific adhesins, or more broadly, act as anti-adhesive substances by covering relevant surfaces. We use whole-cell assays, where we coat host cell ECM molecules to microwell plates and let bacteria bind to them that express the adhesin molecule in question on their cell surface. Such assays may have lower sensitivity and slower response time compared to assays based on purified protein components (Shapiro and Baneyx, 2007). However, there are also advantages: no complex protein purification procedures are necessary, and generally the cost is low since bacteria reproduce easily and quickly (Van Der Meer et al., 2004). Last but not least, the adhesive molecules have their proper orientation when attached on the cell surface, compared to a random protein solution. Based on fluorescence detection utilizing genetically engineered strains, we can detect and quantify the difference between adherent and non-adherent cells. Our assays are fast, scalable and can be employed in high-throughput screening approaches.

## 2 Material and methods

### 2.1 Strains, plasmids and primers used in this study

**Table 1:** List of strains, plasmids or primers used.

Strains, plasmids or primers	Characteristics	Source or reference
*Strains*		
*E. coli* Top10	Wild type/commercial grade	Our lab
*E. coli* BL21DE3	Wild type/commercial grade	Our lab
*E. coli* CC118	Wild type/commercial grade	Our lab
AS43	Top10 a*rsB*::*sfGFP* carried by J23100 constitutive promoter	This work
AS53	AS43 + pASK-IBA2	This work
AS54	AS43 + pASK-IBA2-YadAwt	This work
AS61	Top10 *glmS*::*sfGFP* carried by J23100 constitutive promoter	This work
AS62	AS61 + pASK-IBA2	This work
AS63	AS61 + pASK-IBA2-YadAwt	This work
AS62	AS61 + pASK-IBA2-YadAwt	This work
AS75	Top10 *glmS*::*sfGFP* carried by arabinose inducible promoter	This work
AS76	AS75 + pASK-IBA2-YadAwt	This work
AS89	BL21DE3 *glmS*::*sfGFP* carried by J23100 constitutive promoter	This work
AS90	AS89 + pASK-IBA2-YadAwt	This work

*Plasmids*		
pAS5	Amp^R^, plasmid containing *sfGFP* gene under *ara*BAD promoter, pBR322 origin	This work
pAS7	Amp^R^, plasmid containing *sfGFP* gene under constitutive J23100 promoter, pBR322 origin	This work
pAS9	Amp^R^, FRT-Km^R^-FRT, arsB sites, plasmid containing *sfGFP* gene under constitutive J23100 promoter, R6K γ origin	This work
pBAD-HisA	Amp^R^, empty expression vector, arabinose promoter	ThermoFisher scientific
pASK-IBA2	Amp^R^, empty expression vector used as control	iba-lifesciences
pASK-IBA2-YadAwt	Amp^R^, plasmid containing *YadA* gene from *Y. enterocolitica* (serotype O:8) under tet-inducible promoter, f1 origin	Our lab/gifted by JCL?
pKD46	Amp^R^, gam beta exo under *ara*BAD promoter, repA101^ts^	(Datsenko and Wanner, 2000)
pKD4	Amp^R^, FRT-Km^R^-FRT, oriRγ	(Datsenko and Wanner, 2000)
pCP20	Amp^R^, Cm^R^, FLP^+^, *λ c*I857^+^, *λ p*_R_ Rep^ts^	(Cherepanov and Wackernagel, 1995)
pKIKOarsBKm	Amp^R^, FRT-Km^R^-FRT, arsB sites, R6K γ origin	(Sabri et al., 2013)

*Primers*		
oAS1	GGCTGTTTTGGCGGATGAGAG	Amplify pBAD vector
oAS2	TTGGTAACGAATCAGACAATTGACGGC	Amplify pAS5
oAS3	GGTTAATTCCTCCTGTTAGCCC	Amplify pBAD vector
oAS4	TCTCCATACCCGTTTTTTGGGCTAACAGGAGGAATTAACCATGCGTAAAGGCGAAGAGCTG	Amplify gbAS1
oAS5	GTATCAGGCTGAAAATCTTCTCTCATCCGCCAAAACAGCCTCATCATTTGTACAGTTCATCCATACC	Amplify gbAS1
oAS6	CACTGTACCTAGGACTGAGCTAGCCGTCAATTGGTAACGAATCAGACAATTGACGGC	Amplify pAra-sfGFP
oAS7	CTAGCGAATTCAACCAAACCAGGAGCAATTGATGCGTAAAGGCGAAGAGCTG	Amplify pAra-sfGFP
oAS8	ATGCGTAAAGGCGAAGAGCTG	Amplify pAS5
oAS18	ATGCGTAAAGGCGAAGAGCTGTTC	Amplify J23100-sfGFP
oAS20	CTAGTATTTCTCCTCTTTCTCTAGTAGCTAGCACTGTACCTAGGACTGAGC	Amplify J23100-sfGFP
oAS25	CGAAAAGTGCCACCTGCATC	Amplify pAS9
oAS26	GCCATGGTCCATATGAATATCCTCC	Amplify pAS9
oAS27	TTTTTTCGCTGTTTAAGCCGTCAATTGTCTGATTCGTTAC	Amplify pAS7
oAS28	GGTGCCGGTGGATCGTTCACCGACAAACAACAGATAAAAC	Amplify pAS7
oAS31	CTGTTGTTTGTCGGTGAACGATCCACCGGCACCATCAC	Amplify pKIKOarsBKm
oAS32	AATCAGACAATTGACGGCTTAAACAGCGAAAAAACCCCGC	Amplify pKIKOarsBKm
oAS0111	GTGTAGGCTGGAGCTGC	Amplify pKD4
oAS0112	ATGAATATCCTCCTTAGTTCC	Amplify pKD4
oAS0113	TGCTACTCCGTCAAGCCGTC	Amplify pAS5/pAS7
oAS0114	GAAGCAGCTCCAGCCTACACAGCGTTCACCGACAAACAACAG	Amplify pAS5/pAS7
oAS0116	AGGACAAACAGGTGACAGTTATATG	Amplify gDNA & SOE
oAS0117	GGCGGTCAGTTGTATGTCTTC	Amplify gDNA & SOE
oAS0118	GACGGCTTGACGGAGTAGCATCCATTTATTACTCAACCGTAACC	Amplify gDNA
oAS0137	GGAACTAAGGAGGATATTCATCCTGCGTAAGCGGGGCATTTTTC	Amplify gDNA
oAS121	CACCGAACAACGAATTGCTG	Verify integration
oAS122	CTCCTGGGAGGATTCATAAAGC	Verify integration

### 2.2 Media and growth conditions

We routinely used Luria-Bertani (LB) liquid media (10 g/L Tryptone, 10 g/L NaCl, 5 g/L Yeast extract). For solid medium, 15 g/L agar was added prior to autoclaving for 20 min at 15 psi (1.05 kg/cm^2^). Liquid media and agar plates were supplemented with 100 µg/ml Ampicillin (Amp), 25 µg/ml Kanamycin (Km) or 25 µg/ml Chloramphenicol (Cm) as needed. For cell recovery after transformation, SOC medium was used (5 g/L Yeast extract, 20 g/L Tryptone, 0.58 g/L NaCl, 0.19 g/L KCl, 0.95 g/L MgCl_2_, 1.2 g/L MgSO_4_, 3.6 g/L Glucose).

### 2.3 Chemicals and reagents

All chemicals were purchased from VWR unless stated otherwise. Enzymes were from New England Biolabs unless stated otherwise. Taq and Phusion polymerases were used in 50 µl reaction volumes in standard polymerase chain reactions (PCR) according to manufacturer recommendations. Qiagen products were used to isolate plasmid DNAs, purify PCR products or purify DNA fragments on gel. Oligos and dsDNA fragments were ordered from IDT. Sequencing was performed by GATC. Gelatin from bovine skin (G9391-100G) was purchased from SIGMA-ALDRICH, Human collagen type I (ST-2150-0030) was purchased from Nordic BioSite AS, Collagen I Rat Protein, Tail (A1048301) and Collagen I Bovine Protein (A1064401) were purchased from ThermoFisher Scientific/Gibco. 384-well plates (738-0175, non-binding) were purchased from VWR, 384-well Cell Culture Microplate (781091), 384-well Poly-d-Lysine CELLCOAT® (781946), and black 96-well plates (655076) were purchased from Greiner, and 96-well transparent plates (82.1581) were purchased from SARSTEDT. Pre-coated 96-well plates with collagen type I from rat were from Gibco (A1142803).

### 2.4 Plasmid construction

YadA used in this study was originally cloned from *Yersinia enterocolitica* o:8 WA-314 (Oberhettinger et al., 2011). Plasmid pAS5 (Appendix Fig. 3) was assembled with isothermal assembly (Gibson assembly (Gibson et al., 2009)) following the recommendations in the manufacturer’s manual (NEB). Briefly, 100 ng of PCR amplified vector (commercially available pBAD-HisA from ThermoFisher Scientific) was mixed with a 3-fold molar ratio of PCR-amplified gbAS1 double-stranded DNA (dsDNA) that contained the coding sequence for *sfGFP*. Combined parts were mixed with Gibson assembly master-mix and incubated at 50 °C for 1 h and subsequently transformed using electro-competent *E. coli*. Cells were left to recover in SOC for 1 h at 37 °C with shaking on a tabletop shaker. Cells were plated and antibiotic-resistant clones were validated by colony PCR using Taq polymerase and sent for sequencing for verification. Plasmid pAS7 (Appendix Fig. 3) was assembled with isothermal assembly as before, following the recommendations in the manufacturer’s manual (NEB). Briefly 100 ng of PCR amplified vector (pAS5) was mixed with a 3-fold molar ratio of dsDNA gBlock from IDTDNA (gbAS2) that contained the coding sequence for *sfGFP*, the constitutive promoter (J23100 iGEM Standard Biological Part registry, http://parts.igem.org/Part:BBa_J23100) and the RBS sequence B0034 (iGEM Standard Biological Part registry, http://parts.igem.org/Part:BBa_B0034). Combined parts were mixed with Gibson assembly master-mix and incubated at 50 °C for 1 h and subsequently transformed using electro-competent *E. coli*. Cells were left to recover in SOC for 1 h at 37 °C with shaking on a tabletop shaker. Cells were plated and antibiotic-resistant clones were validated by colony PCR using Taq polymerase and sent for sequencing for verification. Plasmid pAS9 (Appendix Fig. 3) was assembled with isothermal assembly (Gibson assembly (Gibson et al., 2009)) as above following the recommendations in the manufacturer’s manual (NEB). Briefly, 100 ng of PCR amplified pKIKOarsBKm vector (gift from Lars Nielsen & Claudia Vickers (Addgene plasmid # 46766)) was mixed with a 3-fold molar ratio of PCR-amplified pAS7 vector. Combined parts were mixed with Gibson assembly master-mix and incubated at 50 °C for 1 h and subsequently transformed using electro-competent *E. coli* CC118. Cells were left to recover in SOC for 1 h at 37 °C with shaking on a tabletop shaker. Cells were plated and antibiotic-resistant clones were validated by colony PCR using Taq polymerase.

### 2.5 dsDNA cassette for strain engineering

Assembly of the dsDNA cassette used for chromosomal strain engineering in the *glmS* locus was accomplished by overlap extension PCR (Ho et al., 1989), using the primers described in Table 1. Initially individual gene segments were amplified with their corresponding primers (Appendix Fig. 1A) and were used subsequently in a primer-less PCR as templates (Mix A) for the first 10 cycles. Afterwards, Mix B was added containing only the external primers without any additional template DNA for the remaining 25 cycles.

The dsDNA cassette used for chromosomal strain engineering in the *arsB* locus was amplified from plasmid pAS9 using primers described in Table 1 using Phusion polymerase (NEB) according to manufacturers protocol (Appendix Fig. 1B).

### 2.6 Strain construction

Strain AS43 was constructed based on the recombination protocol described by Datsenko and Wanner (2000). *E. coli* cells freshly transformed with pKD46 were selected on appropriate antibiotics plates (Amp) and at a permissive temperature (30 °C) were used to inoculate 15 ml of LB medium supplemented with Amp. The culture was grown with shaking at 30 °C until an OD_600_ of 0.6 was reached. Subsequently arabinose was added to a concentration of 0.2% and the culture was left to shake for another 10 min before a 15 min incubation on ice. Cells were washed twice with ice-cold water and finally resuspended in 200 µl of ice-cold 10% glycerol. At that point, 200 ng of amplified dsDNA product was mixed with the electrocompetent cells and transformed using a 0.1 cm – gap electroporation cuvette at 1.8 kV in a MicroPulser™ electroporator (BioRad). Cells were left to recover by shaking at 37 °C for 1 h before they were plated on selection (Kan) plates and incubated overnight at 37 °C. For plasmid curing, individual colonies were used to inoculate 1 ml of LB supplemented with Kan and grown for 6 h at 42 °C prior to plating on Kan plates. Single colonies were screened for Amp sensitivity to verify the loss of pKD46 plasmid and colony PCR was used to verify the insertion of the expression cassette in the bacterial chromosome. Eventually, correct insertion was validated with sequencing. The Kan/FRT resistance cassette was excised with the help of the pCP20 plasmid, which subsequently was removed by cultivating the culture at non-permissible temperature (42 °C) as described (Datsenko and Wanner, 2000). Single colonies were tested for Amp and Kan sensitivity in order to verify loss of pCP20 plasmid and excision of Kan cassette from the chromosome. Colony PCR was used to verify the correct size of the insert encoding the fluorescent label in the bacterial chromosome.

Strain AS61.3, 75.3 and AS89 were constructed essentially as described before (Bonde et al., 2016). Briefly, using genomic DNA from *E. coli* Top10 as the template, the left and right flanking sequences from the fluorescent protein expression cassette, corresponding to the genomic insertion site sequences were amplified. Using pAS5 or pAS7 as template, the expression cassette encoding for constitutive or inducible expression of *sfGFP* was amplified. Finally, using pKD4 as template, the Kanamycin (Kan) resistance cassette flanked by FRT recognition sites was amplified. All four pieces of amplified DNA were spliced together using SOE-PCR, generating the double-stranded piece of DNA that was used to incorporate the fluorescent tags into the bacterial chromosome. The recombination protocol was based on (Datsenko and Wanner, 2000). *E. coli* cells freshly transformed with pKD46 were selected on appropriate antibiotics plates (Amp) and at a permissive temperature (30 °C) were used to inoculate 15 ml of LB medium supplemented with Amp. The culture was grown with shaking at 30 °C until an OD_600_ of 0.6 was reached. Subsequently arabinose was added to a concentration of 0.2% and the culture was left to shake for another 10 min before a 15 min incubation on ice. Cells were washed twice with ice-cold water and finally resuspended in 200 µl of ice-cold 10% glycerol. At that point, 200 ng of SOE product was mixed with the electrocompetent cells and transformed using a 0.1 cm – gap electroporation cuvette at 1.8 kV in a MicroPulser™ electroporator (BioRad). Cells were left to recover by shaking at 37 °C for 1 h before they were plated on selection (Kan) plates and incubated overnight at 37 °C. For plasmid curing, individual colonies were used to inoculate 1 ml of LB supplemented with Kan and grown for 6 h at 42 °C prior to plating on Kan plates. Single colonies were screened for Amp sensitivity to verify the loss of pKD46 plasmid and colony PCR was used to verify the insertion of the expression cassette in the bacterial chromosome. Eventually, correct insertion was validated with sequencing. The Kan/FRT resistance cassette was excised with the help of the pCP20 plasmid, which subsequently was removed by cultivating the culture at non-permissible temperature (42 °C) as described (Datsenko and Wanner, 2000). Single colonies were tested for Amp and Kan sensitivity in order to verify loss of pCP20 plasmid and excision of Kan cassette from the chromosome. Colony PCR was used to verify the correct size of the insert encoding the fluorescent label in the bacterial chromosome, and sequencing was used to validate the correct sequence of the insert.

### 2.7 Assay optimization – integration sites

In order to assess the influence of the locus of incorporation of our reporter gene, we used overnight pre-cultures (of strains AS53, AS54, AS62, AS63), which were diluted 100-fold. After cultivation under shaking at 37 °C for 3 h, expression of *YadAwt* (from *Y. enterocolitica* serotype O:8) situated on pASK-IBA2 plasmid was induced. The same approach was followed for an empty control vector (pASK-IBA2). For both cases, part of the culture was removed before induction that served as not-induced control and was further cultivated with shaking at 37 °C for 3 h.

Strains AS53 and AS54 have as insertion site the *arsB* locus whereas strainsAS62 and AS63 have *glmS* as insertion site in the bacterial chromosome. While the expression of YadAwt was underway (with shaking at 37 °C for 3 h), microplate wells (96-well format) were coated with 100 µl of 10 µg/ml bovine collagen type I in 0.01 M acetic acid for 30 min. After collagen was removed, wells were blocked with 100 µl of 50 mg/ml BSA in PBS and incubated at RT for 45 min. BSA was removed and cells washed 3 times with 100 µl PBS. Bacterial cell cultures was normalized to OD_600_ of 0.2 using PBS and 100 µl of bacterial cell culture was dispensed on the collagen pre-coated plate and left for 30 min incubation. Cell culture was removed and washed twice with 100 µl of PBS and finally 100 µl of PBS was dispensed and fluorescence was recorded (exc. 488 nm/em. 533 nm).

### 2.8 Assay optimization – collagen

In order to assess the influence of different collagen coating concentrations and sources overnight pre-cultures (strain AS63 expressing *sfGFP* constitutively or strain AS76 expressing *sfGFP* under arabinose promoter) were used, which were diluted 100-fold and after cultivation under shaking at 37 °C for 3 h, before expression of *YadAwt* encoded on the pASK-IBA2 plasmid was induced The same induction protocol was followed for a control vector (only pASK-IBA2). In the case of the AS76 strain, growth media contained 0.01% arabinose to ensure *sfGFP* expression. Assay setup is the same as in Section 2.7 with the following modifications: microplate wells were coated with 100 µl of 10 to 200 µg/ml bovine collagen type I in 0.01 M acetic acid for 30 min or 100 µl of 10 µg/ml of different types of collagen.

### 2.9 Assay setup for high-throughput screening (HTS)

A single colony of AS75.3 freshly transformed with pASK-IBA2-YadAwt was used to inoculate 2.5 ml of LB supplemented with 100 µg/ml Amp and 0.01% Arabinose. The pre-culture was incubated overnight at 37 °C with shaking (200 rpm). A 1:100 dilution was carried-out in 200 ml of fresh medium. The bacterial culture was incubated at 37 °C with shaking (200 rpm) until an OD_600_ of ∼0.6–0.8. 10 ml was removed to serve as uninduced control and was further cultivated with shaking at 37 °C for 3 h, and to the remainder of the culture 19.2 µl of 1 g/L anhydrotetracycline was added for induction of YadA expression for 3 h. Post-induction, OD_600_ was measured and both induced and uninduced control cultures were diluted to and OD_600_ of 0.25 using sterile PBS as diluent. In the meantime, 384-well microplates were coated with 100 µg/ml (in 0.01 M acetic acid) Collagen type I from Bovine source for 30 min. After collagen was dispensed, the microwell plates were centrifuged shortly (15 s pulse using a Centrifuge 5810R from Eppendorf). After this, all dispensing steps were performed using a liquid handling robot (EL406 BioTek unless stated otherwise). Following the collagen incubation period, liquid was removed manually and 10 µl of PBS was dispensed into the wells. Screening compounds in various concentrations were dispensed into the wells using an Echo 555 (Labcyte). 40 µl of bacterial suspension was added and plates were incubated at room temperature for 30 min. Following the incubation time, the bacterial suspension was removed and plates were washed 3 times with 50 µl PBS. Finally, 50 µl PBS was dispensed and fluorescence emission was recorded at 533 nm following excitation at 488 nm (BioTek Synergy H).

### 2.10 Compound collection

We screened a chemical diversity library consisting of 28,000 small molecule compounds provided by Enamine and Chembridge, located at the Center for Molecular Medicine Norway (NCMM). The library is preselected to follow Lipinski’s rules for good solubility, absorption, distribution, metabolism and excretion profile (Lipinski et al., 2012).

### 2.11 Theory/calculation

Experiments were conducted at least in triplicate. Error bars in all figures represent the mean value ± standard deviation. Data of dose response experiment (0–200 µM small molecule concentration) was used to calculate IC_50_ (small molecule concentration that causes 50% inhibition of adhesion compared to adhesion in the absence of small molecule inhibitor). Origin (OriginLab, Northampton, MA) was used for statistical evaluations

## 3 Results and discussion

### 3.1 General considerations

To optimally set-up and utilize our assays, several assay parameters had to be considered. One of them was the decision between a cell-based system or a purified-protein system. While protein-based assays tend to be more reproducible (i.e. typically yield results with better statistical parameters/smaller error bars), cell-based assays approximate the *in vivo* conditions of YadA-based adhesion better. YadA forms two-dimensional arrays on the bacterial cell surface that are not present when the protein is in solution (Hoiczyk et al., 2000).

An adhesin-coated surface would most likely not have all proteins oriented properly and would again not mimic the *in vivo* arrangement properly. In addition to that, expressing and purifying the full-length YadA protein is laborious and would most likely lead to variations from batch to batch. An alternative to this approach would have been to express only parts of the protein, more relevant to the binding to the ECM component, but then again this system would not approximate the oriented *in vivo* conditions.

For the binding partner for YadA, we had to choose between purified collagen or utilizing a mammalian cell line. Our decision was to use collagen, since it is commercially available, easy to handle and in comparison to a mammalian cell line, and does not require any particular maintenance conditions. The added expenses for equipment needed to maintain and handle the cell line were also a factor that was taken into account.

Finally yet importantly, in order to obtain better sensitivity for our assay setup we decided to use fluorescence and not absorbance as a readout. Fluorescent labeling of the cells by genetic manipulation was preferred over later staining protocols to avoid additional dispensing and washing steps in the HTP protocol. Green fluorescent protein (GFP) from *Aequorea Victoria* is frequently utilized as a reporter protein because it is fluorescent without the need of external substrates or cofactors, has low toxicity, and is thermally and chemically stable (Waldo et al., 1999). We decided to use superfolder GFP (sfGFP) protein in our experiments, due to its superior characteristics with regards to folding kinetics, resistance to random mutations and chemical denaturants (Pédelacq et al., 2006). We chose *E. coli* as the host for GFP manipulations and for expression of our target adhesin, as this will allow us to use multiple adhesins from different sources in an identical genetic background in the future. Adhesion is a easy-to-monitor readout for expression of intact adhesins on the cell surface. Compounds identified in this standardized screen can then later be tested with the original pathogens.

### 3.2 Optimization of the fluorescent expression strain

**Fig. 1:**
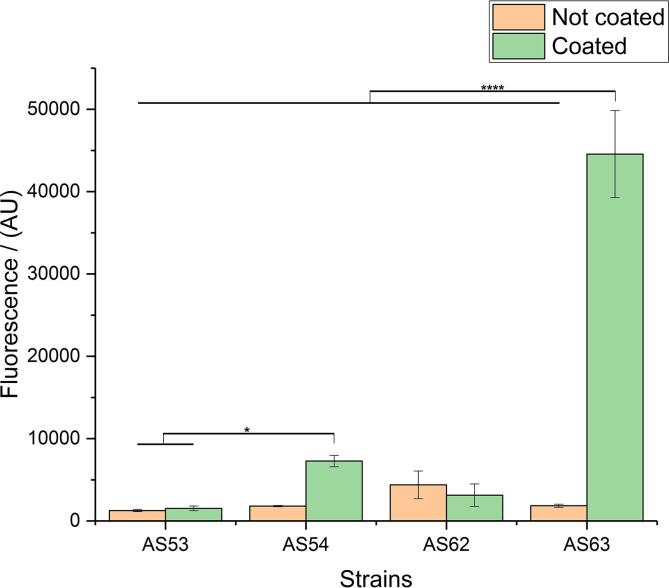
Influence of genomic location of the *sfGFP* gene on the assay. Strain 53 has *sfGFP* integrated in the genome (at the *arsB* locus) under a constitutive promotor. In addition, the strain carries the pASK-IBA2 plasmid (empty vector control). Strain 54 is the same as strain 53 (*sfGFP* integrated in the genome at the *arsB* locus under a constitutive promotor), but carries the pASK-IBA2-YadAwt plasmid. Strain 62 has *sfGFP* integrated in the genome at the *glmS* locus under a constitutive promotor and carries the pASK-IBA2 plasmid (empty vector control). Strain 63 is the same as strain 62 but carries the pASK-IBA2-YadAwt plasmid. Coating of wells was performed with 10 µg/ml bovine collagen type I in 0.01 M acetic acid. YadA expression was induced with 0.2 µg/ml ATc. Data presented as means and standard deviations (SD) (*n* = 4). Asterisks indicate significant differences between compared groups using ANOVA, P < 0.05. Tukey's multiple comparisons indicated. (^****^p ≤ 0.0001, ^***^p ≤ 0.001, ^**^p ≤ 0.01, ^*^p ≤ 0.05, not significant statistical difference is p > 0.05 and is not depicted).

Plasmid-based expression systems allow for multiple gene copies per single cell that in the case of GFP as a reporter results in increased fluorescence of the cells. For this approach, selection markers need to be present at all times to avoid loss of the fluorescent phenotype, which can be challenging when combined with other plasmids for expressing target proteins of a screen. A single copy of the *sfGFP* gene in the bacterial genome effectively prevents such a loss and provides a more reliable and robust strain for a high-throughput setup. We decided to generate a robust screening system by integrating the reporter gene in the bacterial genome. We used the same approach as described recently in literature (Bonde et al., 2016, Sabri et al., 2013) to generate our reporter strain for constitutive sfGFP expression and based on the same methodology, also engineered a strain for inducible sfGFP expression. We transformed these strains with the plasmid containing *YadAwt* (from *Y. enterocolitica* serotype O:8) or the control empty vector and expression was induced by addition of ATc. Fig. 1 shows the performance of different sfGFP-expressing strains. All strains used in this experiment were expressing *sfGFP* constitutively. The chromosomal locus of the insertion of the *sfGFP* gene significantly influences performance.

We chose genomic sites that have been used successfully in the past for the integration of fluorescent proteins (Bonde et al., 2016, Sabri et al., 2013). We observed that the *glmS* integration site construct displays 6-fold increase in relative fluorescence compared to the *arsB* site. We assume that this is based on differences in transcription efficiency. In addition to that and as anticipated, there is much stronger adhesion of bacterial cells expressing YadA to collagen as compared to the non-coated wells. For strain AS54 we observed 4-fold increase of adhesion in the coated surface and for strain AS63 24-fold increase.

### 3.3 Optimization of inducer levels

**Fig. 2:**
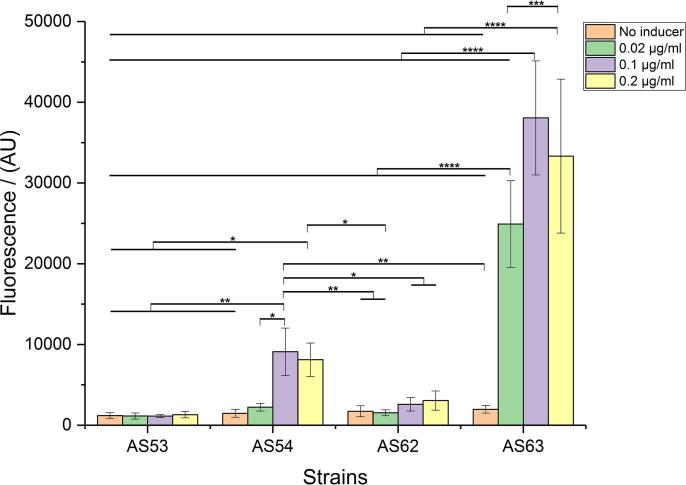
YadA expression and adhesion. The strains used are the same as in Fig. 1. Coating of wells was performed with 10 µg/ml bovine collagen type I in 0.01 M acetic acid. Inducer used was anhydrotetracycline (ATc) at concentration of 0–0.2 µg/ml. Data presented as means and standard deviations (SD) (*n* = 8). Asterisks indicate significant differences between compared groups using ANOVA, P < 0.05. Tukey's multiple comparisons indicated. (^****^p ≤ 0.0001, ^***^p ≤ 0.001, ^**^p ≤ 0.01, ^*^p ≤ 0.05, not significant statistical difference is p > 0.05 and is not depicted).

To optimize adhesin expression, we varied inducer concentrations. The plasmid backbone manufacturer (IBA) recommends 0.2 µg/ml anhydrotetracycline (ATc) as the inducer concentration. ATc is used for induction of the tet promoter-controlled pASK-IBA vectors. ATc is a derivative of tetracycline that exhibits no antibiotic activity when using the manufacturer’s recommended concentrations (https://www.iba-lifesciences.com/details/product/9.html). We used the same experimental conditions and strains as in Fig. 1 in this experiment, but varied the ATc concentration from 0 to 0.2 µg/ml. While carrying out our assay we observed autoaggregation, a known effect of YadA overexpression (Grosskinsky et al., 2007), or due to stress-mediated growth defect or even cell death under extreme overexpression conditions. Under conditions that lead to autoaggregation, monitoring optical density OD_600_ for normalization purposes can be tedious. Based on the results in Fig. 2 we decided to use 0.1 µg/ml as inducer concentration for the rest of the studies.

### 3.4 Optimization of collagen coating

**Fig. 3:**
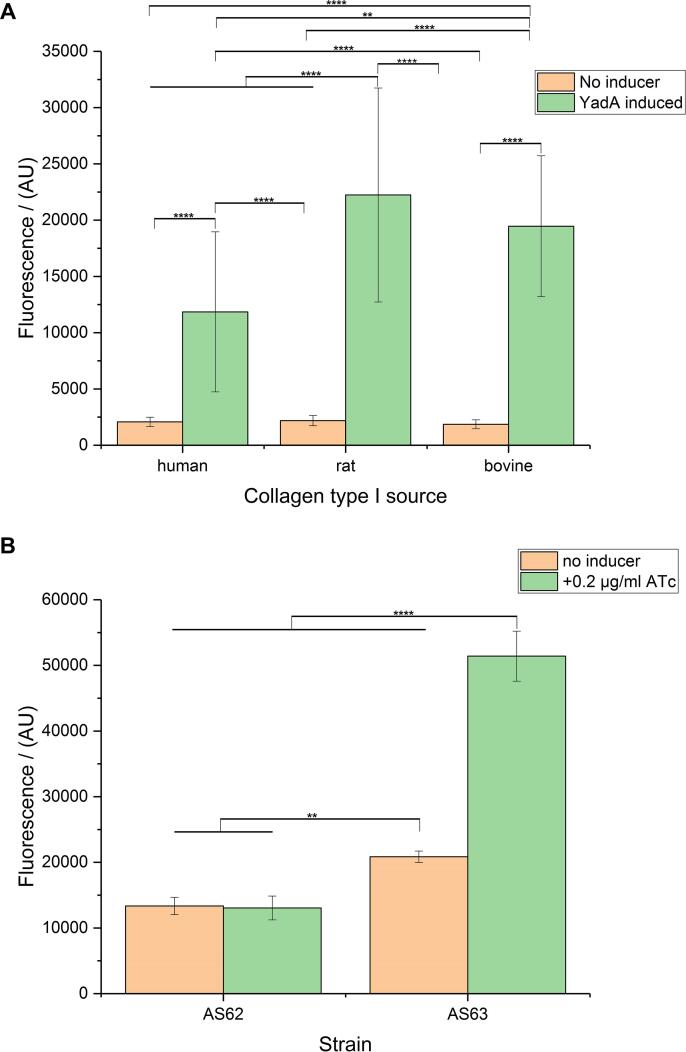
A. Effect of various types of type I collagen on the assay. Strain 63 was used in this experiment, carrying either the empty vector or the plasmid for expressing YadA. Collagen type I from different sources was used at coating concentrations of 10 µg/ml. YadA expression was induced with 0.1 µg/ml ATc. Data presented as means and standard deviations (SD) (*n* = 16). B. Testing pre-coated plates. Rat collagen type I pre-coated 96-well plates (GIBCO) were used in this experiment. The inducer concentration was 0.2 µg/ml anhydrotetracycline (ATc). Data presented as means and standard deviations (SD) (*n* = 4). Asterisks indicate significant differences between compared groups using ANOVA, P < 0.05. Tukey's multiple comparisons indicated. (^****^p ≤ 0.0001, ^***^p ≤ 0.001, ^**^p ≤ 0.01, ^*^p ≤ 0.05, not significant statistical difference is p > 0.05 and is not depicted).

When we investigated the impact of different sources of collagen type I (Fig. 3A), we observed that all collagen type I sources (rat, bovine and human) display similar binding of YadA-expressing cells based on statistical significance analysis. We chose bovine collagen type I for further experiments based on cost and regional availability considerations.

As an alternative option, plates pre-coated with collagen by the manufacturer are available. We investigated the applicability of commercially available rat collagen pre-coated plates in our initial assay optimization steps, hoping that this would yield results with higher reproducibility for high-throughput screening. We observed an almost 2.5-fold difference between the induced and the not-induced control (for YadA wt) and small statistical errors within the measurement (Fig. 3B). However, we decided not to pursue this option further as the HTS setup would ideally require the use of 384-well plates (our results are from 96-well plates and 384-well precoated plates are not available at a price that was feasible for us in high-throughput situations).

### 3.5 Further optimization of the assay

**Fig. 4:**
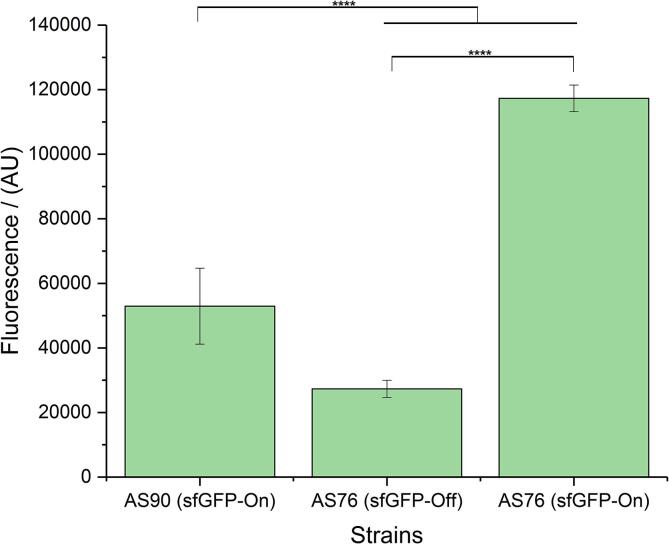
Fluorescence profiles of reporter strains expressing sfGFP in different genomic backgrounds. Strain 76 has *sfGFP* integrated in the genome of *E. coli* Top10 and its expression is under the control of arabinose promoter – induced with 0.01% arabinose and carries the pASK-IBA2-YadAwt plasmid. Strain 90 has *sfGFP* integrated in the genome of *E. coli* BL21(DE3) under constitutive promoter and carries the same pASK-IBA2-YadAwt plasmid. Data presented as means and standard deviations (SD) (*n* = 9). Asterisks indicate significant differences between compared groups using ANOVA, P < 0.05. Tukey's multiple comparisons indicated. (^****^p ≤ 0.0001, ^***^p ≤ 0.001, ^**^p ≤ 0.01, ^*^p ≤ 0.05, not significant statistical difference is p > 0.05 and is not depicted).

For more flexibility in the assay, and to achieve more control over reporter protein levels and turnover, we generated two additional strains. Both strains harbor the reporter gene in the same genomic context (*glmS* locus), however one strain is based on *E. coli* Top10 and has an Arabinose-inducible promotor (AS76) whereas the other strain is based on *E. coli* BL21(DE3) and has constitutive reporter gene expression as described above (AS90). *E. coli* BL21 and its DE3 derivative is a standard laboratory strain used regularly for recombinant protein expression (Jeong et al., 2015, Studier and Moffatt, 1986), while arabinose-inducible protein expression is a commonly used, well-characterized and simple mechanism with high levels of induced expression and low levels of uninduced expression (Schleif, 2000). Under our experimental conditions we observed that the fluorescence levels of the inducible reporter strain were higher (2.2-fold) compared to the strain with constitutive gene expression (Fig. 4). For the following experiments we thus decided to continue with strain AS76.

### 3.6 Blocking conditions

Having decided on the strain to use in the following experiments (AS76) we wanted to investigate the influence of parameters such as coating and blocking of wells, different collagen concentrations and different collagen form.

**Fig. 5:**
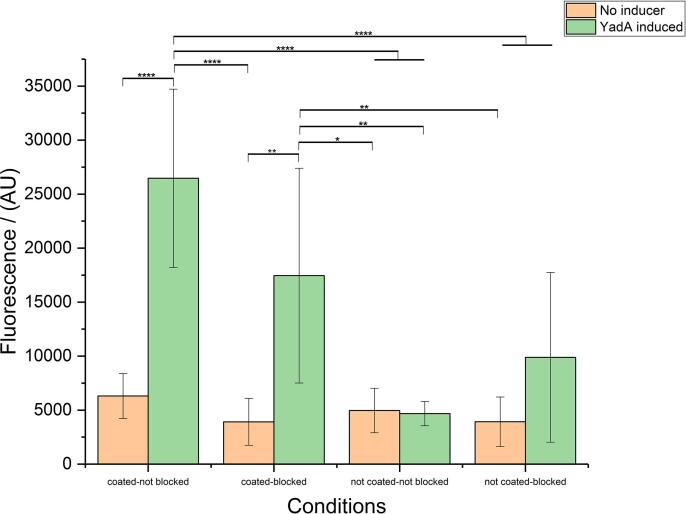
Impact of coating and blocking on the binding of YadA-expressing bacteria. Strain 76 carries the pASK-IBA2-YadAwt plasmid – induced with 0.1 µg/ml ATc and has *sfGFP* integrated in the genome and its expression is under the control of an arabinose-inducible promoter – induced with 0.01% arabinose. Coating was done with 100 µg/ml bovine collagen type I for 30 min, blocking was done with 50 mg/ml BSA in PBS for 30 min. Data presented as means and standard deviations (SD) (*n* = 6). Asterisks indicate significant differences between compared groups using ANOVA, P < 0.05. Tukey's multiple comparisons indicated. (^****^p ≤ 0.0001, ^***^p ≤ 0.001, ^**^p ≤ 0.01, ^*^p ≤ 0.05, not significant statistical difference is p > 0.05 and is not depicted).

Blocking is a step that is used to prevent high background of cells binding directly to the plastic surfaces of the wells. In order to transfer our pilot assays to the HTS setup we investigated the option of blocking of wells with different protocols using BSA after applying the collagen coating. However, under our experimental conditions we observed a better signal to noise ratio between cells expressing the adhesin YadA and the uninduced control cells for the collagen-coated but not BSA-blocked wells, compared to the experiment with BSA-blocked wells (4.4- compared to 4.1-fold change respectively) (Fig. 5). In order to minimize the number of pipetting steps and therefore the assay duration, and to eliminate steps that create potential complications with the HTS instrumentation, we decided not to block our wells with BSA for the HTS setup.

### 3.7 Coating conditions

**Fig. 6:**
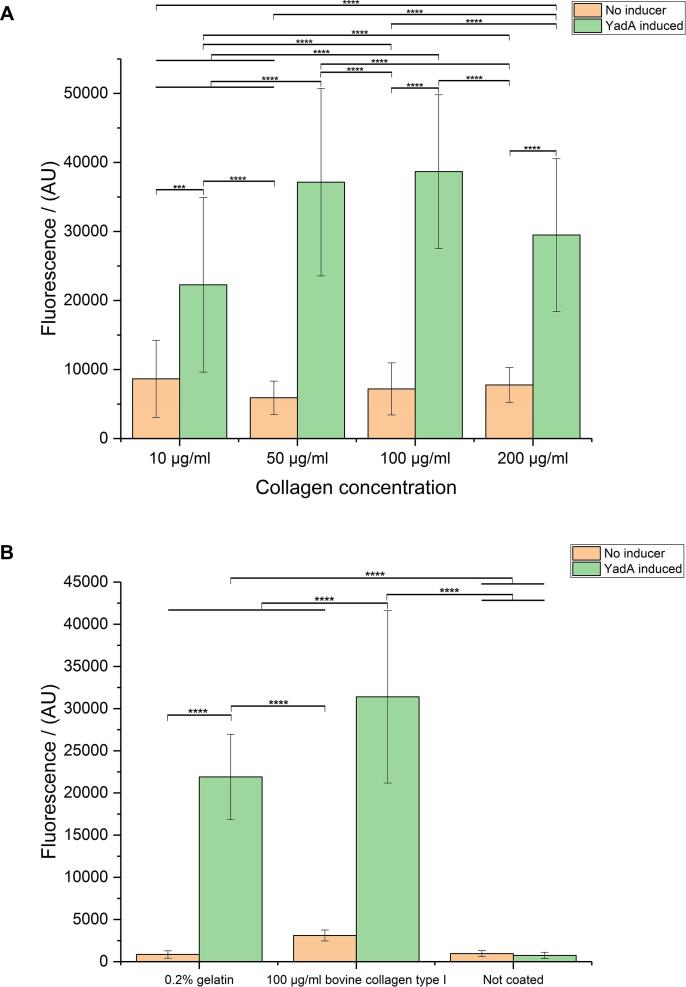
A. Effect of different concentrations of bovine collagen type I in the coating process. Strain 76 has *sfGFP* integrated in the genome and its expression is under the control of arabinose promoter – induced with 0.01% arabinose, and carries the pASK-IBA2-YadAwt plasmid. Coating was performed for 30 min. 0.1 µg/ml ATc was used for YadA expression. B. Comparison of bovine gelatin to type I bovine collagen in the YadA binding assay. Strain 76 has *sfGFP* integrated in the genome and its expression is under the control of arabinose promoter – induced with 0.01% arabinose and carries the pASK-IBA2-YadAwt plasmid. 0.1 µg/ml ATc was used for YadA expression. Data presented as means and standard deviations (SD) (*n* = 16). Asterisks indicate significant differences between compared groups using ANOVA, P < 0.05. Tukey's multiple comparisons indicated. (^****^p ≤ 0.0001, ^***^p ≤ 0.001, ^**^p ≤ 0.01, ^*^p ≤ 0.05, not significant statistical difference is p > 0.05 and is not depicted).

To ensure that our collagen coating procedure is optimal, we investigated the impact of the collagen concentration. When we used a range of 10–200 µg/ml of bovine collagen type I to coat the wells of microtiter plate suitable for the HTS (384× wells) we observed that collagen concentrations of 10 µg/ml as we used previously generated data that had unacceptable signal to noise performance. When using 50–200 µg/ml we obtained the best signal to noise ratios when compared to 10 µg/ml collagen coating concentrations. At the end, we decided to use a coating concentration of 100 µg/ml collagen for future experiments (Fig. 6A).

At a later stage when we were ready to transfer our pilot assays to the HTS setup we contemplated using gelatin from bovine skin as a plate coating material in order to reduce costs and generate a more even, smoother coating layer compared to collagen. Gelatin is composed of partially denatured collagen and other ECM compounds. Gelatin solutions are liquid when heated and gel-like when cooled down, which in principle would allow an evenly coated surface in the wells, potentially more even than random deposition of collagen fibers. The experiment in Fig. 6B shows that gelatin can be used for coating and that YadA binds to gelatin. However, when we wanted to combine gelatin coating with our HTS setup this proved to be rather cumbersome, as the material needs to be kept at higher than 37 °C for dispensing purposes and the liquid handling robot we wanted to use did not allow for this temperature in a stable way. For practical reasons we decided to continue with coating with collagen instead of gelatin.

### 3.8 Development of a high-throughput workflow

**Fig. 7:**
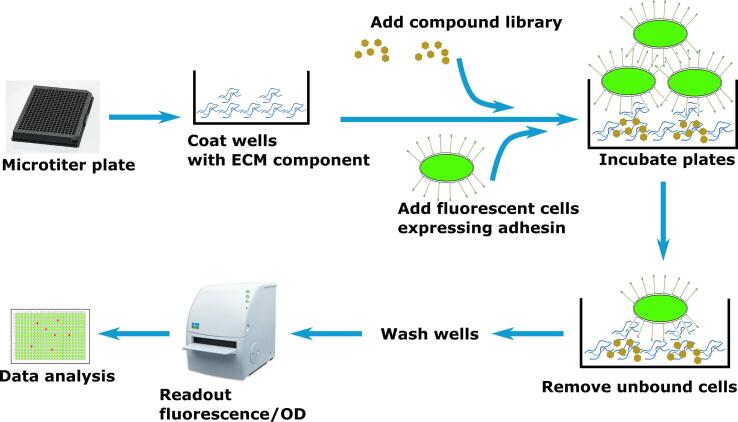
Screening workflow. Microtiter plates are coated with the ECM component (collagen), subsequently compound library is added onto the plates, followed by addition of bacterial cells expressing both the fluorescent reporter and the adhesin (YadA). After incubation, removal of unbound cells and washing of the wells, fluorescence is recorded with the help of a plate reader. All steps are performed by liquid handling robots except collagen dispensing and removal.

A small-molecule inhibitor screening workflow was developed based on the results above, using whole bacterial cells that express YadA, collagen-coated 384-well plates, and a library of 28,000 compounds. The workflow of the assay is displayed in Fig. 7.

From all compounds screened at a standard concentration of 10 μM, the best 514 initial hits were chosen to check assay reproducibility. These hits showed anti-adhesive effects which was determined by a statistical cutoff of three standard deviations below the mean of the positive control. We repeated the adhesion assay in triplicates for these 514 compounds. From these, we selected 16 that were fully reproducible and performed to a level of inhibition <80% adhesion compared to the positive control (i.e. strain 76 carrying pASK-IBA2-YadAwt plasmid where expression of *sfGFP* and *YadA* are induced). On these 16 hits, we performed dose-response experiments in a range from 0 to 20 µM (Appendix Fig. 4). Out of the 16 compounds tested in dose response experiments, 4 were eliminated due to intrinsic fluorescence properties. From the remaining 12 compounds, 10 showed at least 25% inhibition of adhesion at 10 μM and 4 out of the 12 showed at least 50% inhibition of adhesion (i.e. <50% of residual adhesion) at 20 μM.

Finally, in our last experiment we wanted to demonstrate the broader dose -dependent effect for 5 of the compounds that reproducibly demonstrated inhibition of bacterial adhesion, therefore we tested compound end concentrations ranging from 0 to 200 µM. At the same time, we tested two different plate types to rule out plastic-specific side effects.

We used two different type of plates from the same manufacturer, one cell-culture grade and one cell-culture grade with poly-d-lysine pre-coating. Moreover, we left part of the plate not coated with collagen as a background control. In order to investigate the effect of DMSO in the assay we dispensed only DMSO in some of the wells, in amounts equivalent to the DMSO amounts dispensed with increasing compound volumes.

**Fig. 8:**
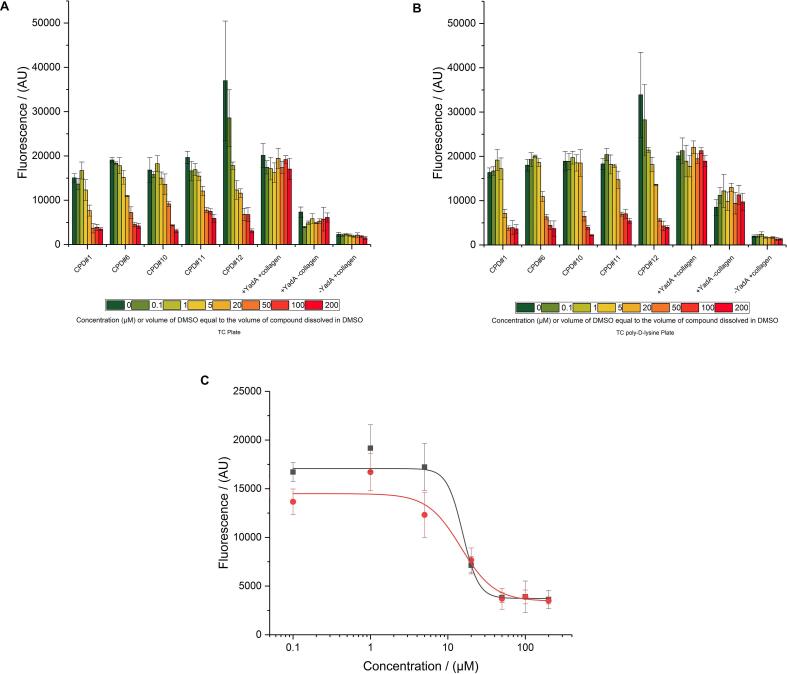
Dose-dependency of adhesion inhibition. For 5 compounds identified as hits, concentration ranges from 0 to 200 µM (dissolved in DMSO) were tested. The controls contained only DMSO in increasing volume, to correspond to the increasing volume of compound dissolved in DMSO to rule out solvent effects. Highest concentration of DMSO is 2%. Control: +YadA + collagen is strain 76 carrying the pASK-IBA2-YadAwt plasmid where YadA expression is induced, Control: −YadA + collagen is the same as Control: +YadA + collagen but YadA expression is not induced. For Control: +YadA − collagen we used YadA expressing bacteria in a region of the plate that we did not coat with collagen. The rest of the plate was coated with 100 µg/ml collagen prior to dispensing the compounds or DMSO. Plate types: A (left panel) – cell culture plate B (right panel) – cell culture plate, poly-d-lysine coated plate. 0.1 µg/ml ATc was used for YadA expression C. Dose-response curve for compound 1. Stain AS76 carrying the pASK-IBA2-YadAwt plasmid where expression is induced, was incubated with increasing concentrations of compound 1. The reduction of adhesion was measured as a reduction of fluorescence signal. Compound 1 inhibits adhesion with an IC50 of 15.5 ± 4.9 µM for the tissue culture grade plate or 14.7 ± 4 µM for the tissue culture grade plate with poly-d-lysine precoating. (Red circles – Tissue culture grade plates, Black squares – Tissue culture grade plates with poly-d-lysine pre-coating). (For interpretation of the references to colour in this figure legend, the reader is referred to the web version of this article.)

From our broad dose response experiment, we observed an increased inhibition of bacterial adhesion as the compound concentration increased, in a range from 0 to 200 µM (Fig. 8A/B). In order to check the effect of the different volumes of DMSO used to dispense compounds at different amounts on the results and errors of measurement, we decided to use increasing volumes of DMSO for the control wells: +YadA + collagen, +YadA − collagen, −YadA + collagen.. Control: +YadA + collagen is strain 76 carrying the pASK-IBA2-YadAwt plasmid where YadA expression is induced, Control: −YadA + collagen is the same as Control: +YadA + collagen but YadA expression is not induced. For Control: +YadA -collagen we used YadA expressing bacteria in a region of the plate that we did not coat with collagen. The typical screening concentration is 10 µM for each compound, and with the automation setup we used (Labcyte ECHO – acoustic dispensing), this corresponds to a transfer of 50 nl in a total volume of 50 µl, or 0.1% final DMSO concentration. Particularly, for the highest concentration of 200 µM, it would correspond to 1000 nl being transferred in a total volume of 50 µl, or 2% final DMSO concentration which is below cytotoxic concentration (Hakura et al., 1993). For the control experiments we did not observe a decrease of adhesion at the highest DMSO concentrations (Fig. 8A/B). This demonstrates that the inhibitory effects were solely based on our hit compounds and not on the solvent. Another important observation in this experiment is that the plate type affects the background binding of the adhesin-expressing bacteria. This is best seen when comparing wells without collagen coating (control: +YadA − collagen), where we noticed a higher adhesion of TAA-expressing bacteria onto the cell-culture grade plates pre-coated with poly-d-lysine, compared to the standard cell-culture grade plates (Fig. 8A/B). This is not an unexpected finding, as the poly-d-lysine treated plates are designed by the manufacturer so that they generally exhibit higher binding properties to cells than the non-coated ones. Nonetheless, when we dispensed 50–200 µM for compound 1 in both plate types, we observed inhibition of bacterial adhesion down to the level of bacteria where the adhesin expression was not induced (control: −YadA + collagen). This clearly demonstrates the efficacy of the compound as an inhibitor of the interaction between the adhesin YadA and its binding partner, collagen.

For the compound that exhibited the highest inhibition potential we calculated IC_50_ (small molecule concentration that causes 50% inhibition of adhesion compared to adhesion in the absence of small molecule inhibitor) using the data collected from the 0 to 200 µM dose-response experiment (Fig. 8C). Depending on the different plate material used for the assay the values were 15.5 ± 4.9 µM for the tissue culture grade plate or 14.7 ± 4 µM for the tissue culture grade plate with poly-d-lysine precoating.

## 4 Conclusions

We genetically engineered a fluorescent *E. coli* reporter strain, heterologously expressed a bacterial adhesin from *Yersinia enterocolitica*, YadA, in this strain, and developed an assay to quantify bacterial adhesion and inhibition of adhesion in a high-throughput format. This assay was used to interrogate a library of 28,000 compounds. As a result, we obtained 5 hit compounds with the desired functionality that at the highest concentration inhibited the binding of YadA-expressing bacteria to the ECM component collagen to the level of the uninduced control. Unfortunately, the compounds do not show the same inhibitory effect towards wildtype *Yersinia* cells. This is not surprising as the results in *E. coli* are already close to the limit of detection; the compounds will need modifications to improve their binding affinity before we can hope to employ them in any medically meaningful way. The assay presented here should be easy to adapt to other bacterial adhesins that can be expressed on the cell surface of *E. coli*, and to other ECM components, and we hope that this publication gives easy step-by-step explanations of how to set up similar screening assays. This opens the way to the systematic discovery of anti-adhesive molecules that can be further developed into antimicrobial coatings for implants, or drugs to wash out bacteria from the sites of infection. Last but not least, such molecules can be used as tools for a better molecular understanding of the binding surfaces of bacterial adhesins.

## Declaration of Competing Interest

None.
