# Supplementary material for: Assay development for the discovery of small-molecule inhibitors of YadA adhesion to collagen
Source: Cell Surf. 2019 May 23;5:100025. doi: 10.1016/j.tcsw.2019.100025 (PMC7388967; doi:10.1016/j.tcsw.2019.100025)
Supplement: Supplementary data 2 [file mmc2.pdf]

6. Appendices

gbAS0001

atgcgtaaaggcgaagagctgttcactggtgtcgtccctattctggtggaactggatggtgatgttaacggtcataagttttccgtgcgtggcgagggtg
aaggtgacgcaactaatggtaaactgacgctgaagttcatctgtactactggtaaactgccggtaccttggccgactctggtaacgacgctgacttatg
gtgttcagtgctttgctcgttatccggaccatatgaagcagcatgacttcttcaagtccgccatgccggaaggctatgtgcaggaacgcacgatttccttt
aaggatgacggcacgtacaaaacgcgtgcggaagtgaaatttgaaggcgataccctggtaaaccgcattgagctgaaaggcattgactttaaagaa
gacggcaatatcctgggccataagctggaatacaattttaacagccacaatgtttacatcaccgccgataaacaaaaaaatggcattaaagcgaattt
taaaattcgccacaacgtggaggatggcagcgtgcagctggctgatcactaccagcaaaacactccaatcggtgatggtcctgttctgctgccagaca
atcactatctgagcacgcaaagcgttctgtctaaagatccgaacgagaaacgcgatcatatggttctgctggagttcgtaaccgcagcgggcatcacg
catggtatggatgaactgtacaaatgatga

Sequence corresponding to *sfGFP* sequence as ordered from IDTDNA as gbAS1

gbAS0002

CCTATGCTACTCCGTCAAGCCGTCAATTGTCTGATTCGTTACCAATTGACGGCTAGCTCAGTCCTAGGTACAGTGCT
AGCTACTAGAGAAAGAGGAGAAATACTAGATGCGTAAAGGCGAAGAGCTGTTCACTGGTGTCGTCCCTATTCT

Sequence corresponding to constitutive promoter J23100 and flanking sites (underlined) that match pBAD plasmid backbone and *sfGFP* as ordered from IDTDNA as gbAS2

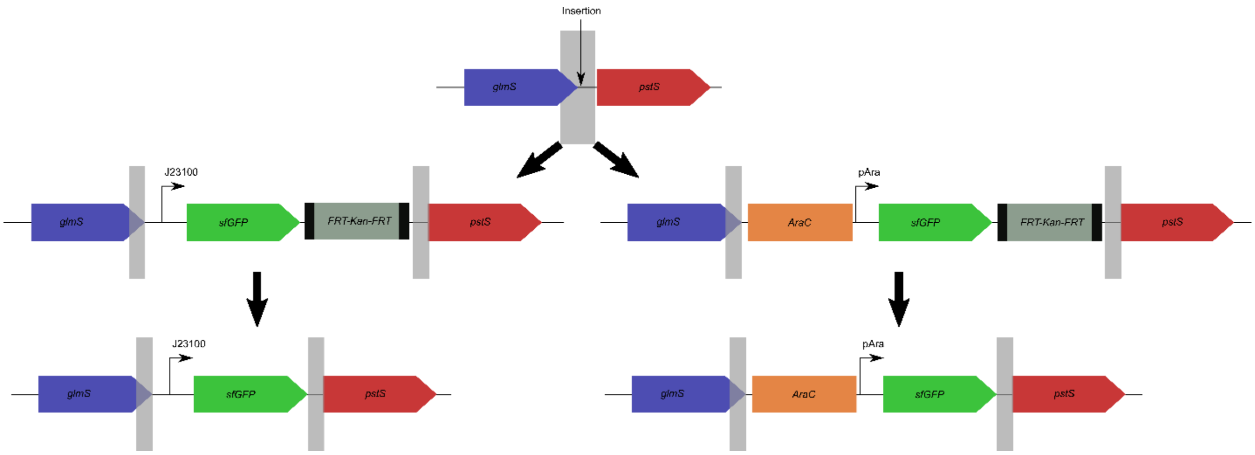

Appendix Figure A1 depicting the step-wise insertion of the reporter protein constructs used in this study, at the *glmS* locus.

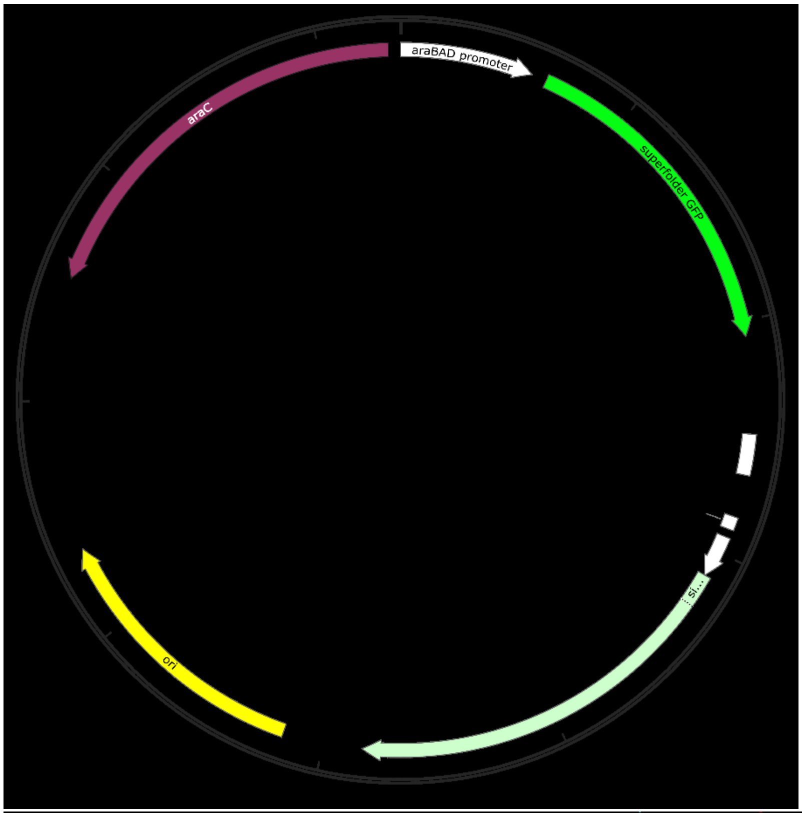
araBAD promoter
araC
superfolder GFP
ori

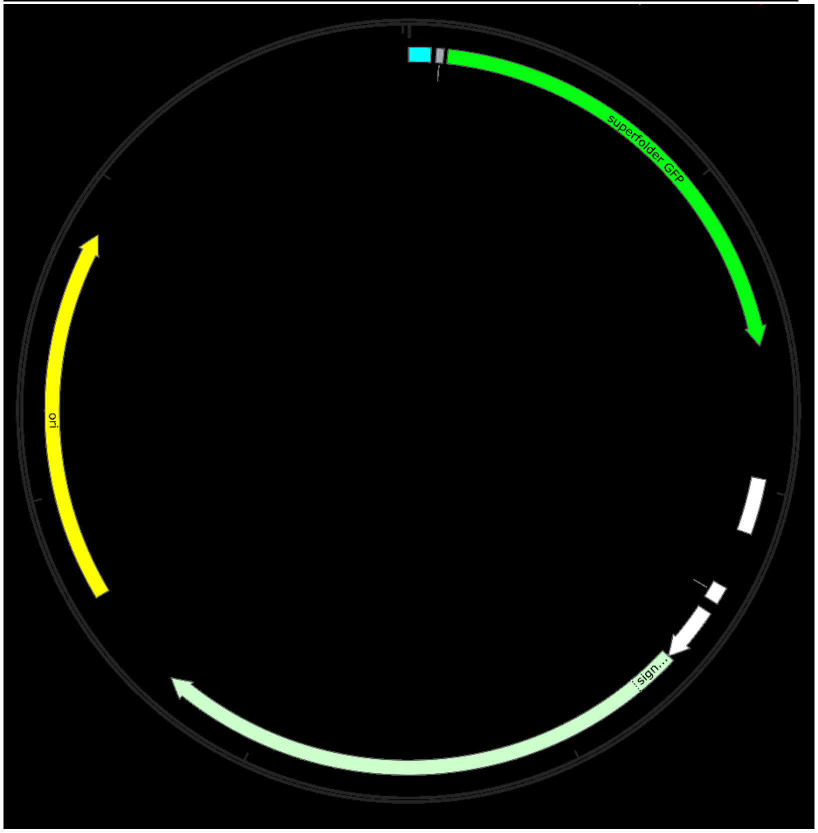
superfolder GFP
ori

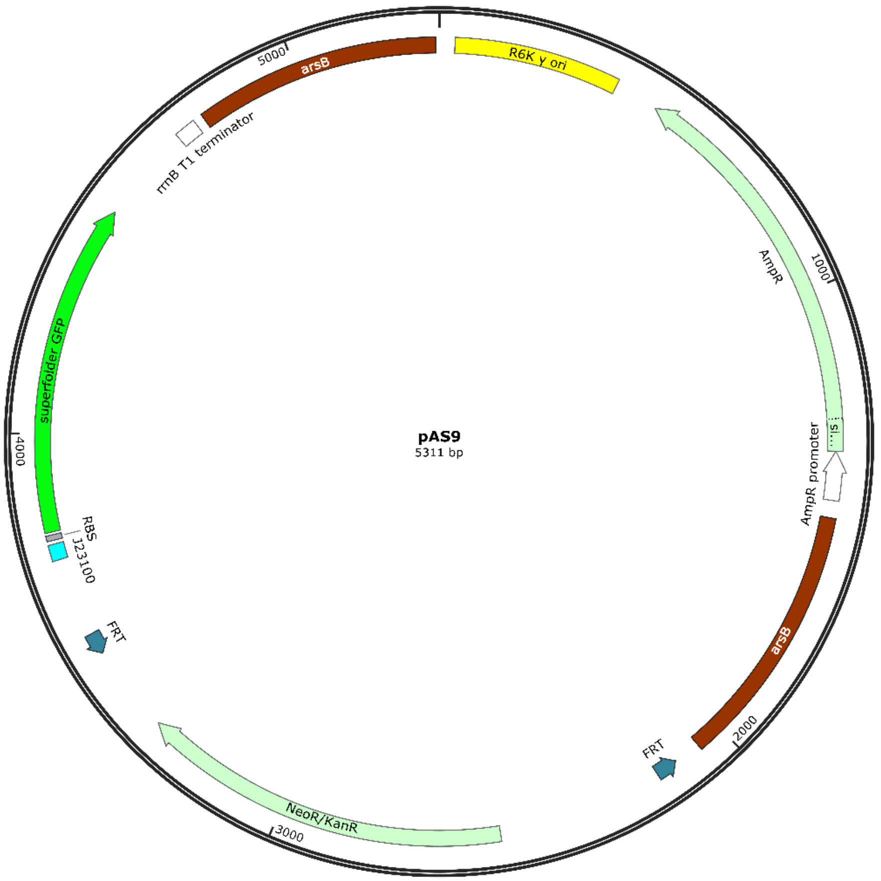

Plasmids pAS5, pAS7, pAS9 used in this study. Plasmid maps generated with SnapGene® software (from GSL Biotech; available at snapgene.com)
